# The silent struggle: anxiety in men with prostate cancer

**DOI:** 10.1093/jncics/pkae094

**Published:** 2024-10-25

**Authors:** Zhiyu Qian, Stephan M Korn, Quoc-Dien Trinh

**Affiliations:** Department of Urology, Brigham and Women’s Hospital, Harvard Medical School, Boston, MA 02115, USA; Center for Surgery and Public Health, Brigham and Women’s Hospital, Harvard Medical School, Boston, MA 02115, USA; Department of Urology, Brigham and Women’s Hospital, Harvard Medical School, Boston, MA 02115, USA; Center for Surgery and Public Health, Brigham and Women’s Hospital, Harvard Medical School, Boston, MA 02115, USA; Department of Urology, Medical University of Vienna, Vienna 1090, Austria; Department of Urology, Brigham and Women’s Hospital, Harvard Medical School, Boston, MA 02115, USA; Center for Surgery and Public Health, Brigham and Women’s Hospital, Harvard Medical School, Boston, MA 02115, USA

The novel study by Crump and colleagues (1) addresses a frequently overlooked issue accompanying prostate cancer—the increased risk of anxiety disorders. The study’s findings, drawn from a vast national cohort from Sweden, paint a stark picture: Men fighting prostate cancer, especially those facing higher-risk or more advanced disease or undergoing androgen deprivation therapy (ADT), are significantly more likely to develop anxiety disorders. This association, most pronounced in the immediate aftermath of diagnosis, casts a long shadow, persisting for years and underscoring the enduring psychological impact of prostate cancer.

The study’s findings underscore the need for a more holistic approach to prostate cancer care. The relatively slower clinical course of prostate cancer, often spanning beyond a decade even in metastatic cases, amplifies the importance of addressing the mental and emotional well-being of patients (2). The protracted nature of the disease, coupled with the potential for long-term treatment side effects, may create a persistent undercurrent of anxiety and uncertainty.

The spotlight on ADT as a contributing factor to anxiety is particularly crucial. Although ADT effectively curbs prostate cancer progression, its side effects, including fatigue, diminished energy, and cognitive decline, can profoundly disrupt a man’s life, especially in older individuals (3). The association between ADT and anxiety may be due to these side effects or to the fact that many men with locally advanced or metastatic disease are dependent on ADT or combination therapies with ADT. The study’s findings highlight the importance of vigilant monitoring and proactive mental health support for men receiving ADT.

Moreover, the landscape of ADT has undergone a transformation. Novel agents targeting multiple steps in the androgen pathway offer promising efficacy but at a steep price (4). The financial toxicity associated with these treatments, such as enzalutamide (Xtandi), which can cost upwards of $15 000 per month in the United States without insurance, adds another layer of distress for men battling advanced prostate cancer. It is noteworthy that this study was conducted in Sweden, a country whose social policies, including comprehensive health and medication coverage, might reduce the financial burdens associated with health care that are often encountered in the United States. Indeed, this financial burden, coupled with the growing trend toward earlier and more aggressive systemic therapies in oncology, can only further exacerbate the financial and mental health challenges faced by these patients in a country such as the United States.

The high prevalence of anxiety disorders in men with prostate cancer, as pointed out by Crump et al. (1), underscores the need for better survivorship care, one that encompasses both the physical and psychological dimensions of the disease. Through proactive screening, early detection, and comprehensive multidisciplinary care that includes psychological expertise when needed, we can empower men with prostate cancer to navigate their journey with enhanced well-being.
